# Effects of microwave blanching conditions on the quality of green asparagus (*Asparagus officinalis* L.) butt segment

**DOI:** 10.1002/fsn3.1199

**Published:** 2019-09-30

**Authors:** Thi Van Linh Nguyen, Thi Yen Nhi Tran, Duc Tri Lam, Long Giang Bach, Duy Chinh Nguyen

**Affiliations:** ^1^ Faculty of Environmental and Food Engineering Nguyen Tat Thanh University Ho Chi Minh Vietnam; ^2^ Center of Excellence for Authenticity Risk Assessment and Technology of Food Nguyen Tat Thanh University Ho Chi Minh City Vietnam; ^3^ NTT Hi‐Tech Institute Nguyen Tat Thanh University Ho Chi Minh City Vietnam; ^4^ Center of Excellence for Biochemistry and Natural Products Nguyen Tat Thanh University Ho Chi Minh City Vietnam

**Keywords:** asparagus butt segment, DPPH, microwave blanching, phenolic degradation

## Abstract

Blanching is a pretreatment method that is often applied in fruit and vegetable processing to inhibit enzyme activity and reduce loss of food quality. It was recently discovered that well‐controlled microwave volumetric heating could improve the blanching efficiency and retain nutritional and sensorial values of product. This study was conducted to investigate effects of microwave blanching conditions on the quality of green asparagus (*Asparagus officinalis* L.) butt segments, a rich source of fiber and antioxidants but are often discarded during processing. The experiments were designed by one‐factor‐at‐a‐time method with two varying factors including blanching time (2, 4, 6, and 8 min) and microwave power output (150, 300, 450, and 600 W). Quality of product was evaluated by sensory, retention of phenolics, and free‐radical scavenging activity retention. The results showed that longer blanching time or higher microwave power was associated with reduced quality of green asparagus butt segment. Besides, the appropriate parameters for microwave blanching of the green asparagus butt segment was found at 300 W for 4 min.

## 1 INTRODUCTION

Green asparagus (*Asparagus officinalis* L) is a vegetable that is rich in vitamins (A, B1, C, E, etc.) and minerals (Mg, Ca, P, Fe) (Joshi, Rawat, Bisht, Negi, & Singh, 2010) and is widely consumed in fresh form in many countries. Some compounds in green asparagus including protodioscin, asparagine, sarsasapogenin, and folic acids have been discovered to confer various health benefits such as anti‐inflammatory, cardiovascular, and anticancer (Shou, Lu, & Huang, 2007). Rutin and phenolic groups are the most important compounds because of their strong antioxidant properties (Nindo, Sun, Wang, Tang, & Powers, 2003; Vinson, Hao, Su, & Zubik, 1998). In the processing of asparagus, only a small proportion of total weight of the plant (2%–3%) is processed into canning (Nindo et al., 2003), while the remainder, which is mostly butt segments, is often discarded due to their high content of cellulose. However, green asparagus butt segments were reported to be abundant with health‐beneficial bioactive compounds such as phytochemicals and fiber (Nindo et al., 2003). Therefore, suitable processing technologies would contribute to the development of economical and healthy products from this potential material.

In processing of fruits and vegetables, blanching process is an important pretreatment technique that was often used to maintain nutritional quality and make the next processing step less time‐consuming and more efficient. Water blanching and steam blanching are common conventional blanching methods that are used widely in the processing industry. However, these techniques require more time and energy to heat the blanching solution to maintain nutrient content and antioxidant ability of the plant materials. Recently, microwave radiation was developed and applied in blanching operation to overcome drawback of conventional methods (Giami, 1991; Ramesh, Wolf, Tevini, & Bognar, 2002; Schirack, Drake, Sanders, & Sandeep, 2006). A comparative study that evaluates impact of hot‐water and microwave blanching technique on quality of green beans suggested that microwave processing of green beans can be a good alternative to conventional blanching methods because of shorter processing times (Ruiz‐Ojeda & Peñas, 2013). Novel blanching treatments such as blanching by high‐humidity hot‐air impingement, microwave, and Ohmic heating could reportedly reduce the nutrition loss and are more efficient in drying (Deng et al., 2017). Even though investigations regarding microwave blanching on different raw materials, such as peanut (Schirack et al., 2006) and lettuce (Wang, Zhang, Mujumdar, Mothibe, & Roknul Azam, 2012), have been reported, there is limitation in study of microwave blanching on asparagus, especially in butt segment of green asparagus.

Given the promising nutritional value of asparagus butt segment and the scarcity of studies related to asparagus blanching, this work aims to investigate effects of microwave blanching conditions on quality of the green asparagus butt segment. The aim of this study is to determine appropriate blanching parameters to retain the high quality of the sample. Quality was determined by indicators including total phenolic content, DPPH radical scavenging capacity, and sensory properties.

## 2 MATERIALS AND METHODS

### 2.1 Materials and Methods

#### 2.1.1 Sample preparation

**Figure 1:**
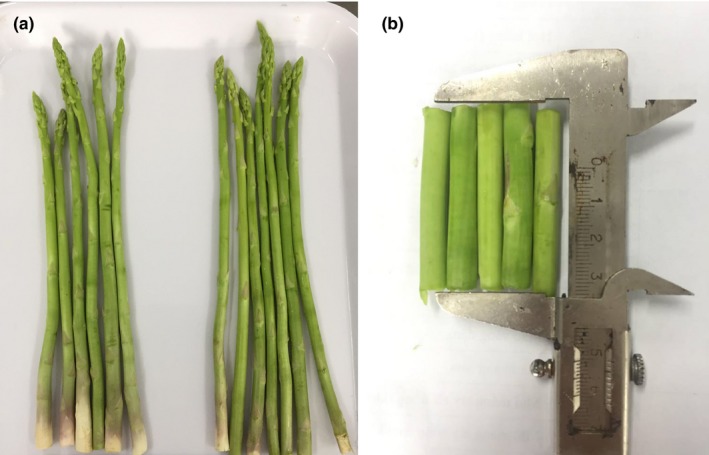
Green asparagus material used in the study (from left to right: materials (a) before cutting, after washing, and (b) after cutting)

Green asparagus spears (*Asparagus officinalis* L.) were purchased at An Phu Dong market, district 12, Ho Chi Minh City, Vietnam. Selected asparagus spears were intact and green. The green asparagus spears were washed. The butt segments were cut into parts, which are 5.0 ± 0.5 cm in length and 0.5 ± 0.2 cm in diameter (Figure 1).

#### 2.1.2 Chemicals

Folin‐Ciocalteu Reagent (FCR), Gallic acid, DPPH (2,2‐diphenyl‐1‐picrylhydrazyl), and Trolox (6‐hydroxy‐2,5,7,8‐tetramethylchroman‐2‐carboxylic acid) were purchased from Sigma‐Aldrich, Co Ltd. (USA), and 2,6‐dichlorophenolindophenol (DCPIP) was imported from India. Other chemicals such as distilled water (with pH between 6.5 and 8), methanol (99.5% purity), Na_2_CO_3_ (99.5% purity), and NaHCO_3_ (99.5% purity) originated from China.

#### 2.1.3 Microwave blanching

First, 10 g of the sample were placed in a pottery bowl and 100 ml of water was added. The bowl was then placed in the microwave oven cavity for blanching. The blanching was performed at four microwave power levels of 150, 300, 450, and 600 W and four duration levels of 2, 4, 6, and 8 min. After blanching, samples were cooled promptly with cold water at 5.0°C ± 0.5°C and then analyzed for total phenolic content and antioxidant capacity.

#### 2.1.4 Analytical method

##### Determination of total phenolic

The total phenolic content was measured by the Folin‐Ciocalteu colorimetric methods, using gallic acid as a standard as described previously (Velioglu, Mazza, Gao, & Oomah, 1998). The blanched sample (5 grams) was ground and extracted with distilled water at the ratio of 1:10. The extract was placed at room temperature about 30 min and then filtered through Whatman No.1. The residue was extracted twice in the same way. The extracts were taken to the norm and analyzed. Extracts (1 ml) were put in a dark tube and added with 1 ml Folin‐Ciocalteu reagent (diluted 10 times with distilled water) and 1 ml sodium carbonate solution (20% w/v). The sample was placed in dark space before being taken to a photometer at an absorption of 765 nm. The total phenolic content was expressed in mg of gallic acid equivalent per gram of dry matter (mg GAE/g dry matter).

##### Determination of antioxidant capacity by DPPH

Antioxidant capacity was measured by scavenging free radical (DPPH), which was performed based on the method described by Braca et al. (2001). Since antioxidant compounds had the ability to scavenge free radicals, discoloration of purple color would occur in the DPPH solution. The blanched sample (5 g) was ground and extracted with methanol. The diluted extract (0.2 ml) was mixed with 3 ml DPPH solution. The sample was kept in the dark for about 30 min and then measured for absorbance at 515 nm. The result was expressed by mg of Trolox equivalent per gram of dry matter (mg TE/g dry matter).

##### Sensory evaluation

After being blanched at different modes, samples will be evaluated with respect to color and texture and compared to the fresh sample. Sensory evaluation was performed by description methods (Li, Zhang, & Yu, 2006). The results were expressed by image and sensory description.

##### Data analysis

All experiments were conducted in triplicate. The mean and standard deviation of the results were calculated using Microsoft Excel program (Microsoft Inc., Redmond, WA, USA). Experiment data were analyzed using one‐way analysis of variance (ANOVA) test in SPSS software (IBM Company) with the level of significance at 5%.

## 3 RESULTS AND DISCUSSION

### 3.1 Investigating asparagus material

Green asparagus materials were analyzed for total phenolic content and antioxidant activity (the DPPH assay). Analytical results were showed in Table 1. The results showed that the total phenolic content and antioxidant activity in butt segment were lower than those in bud segment by 51.50% and 52.26%, respectively.

**Table 1:** Comparison of phenolic content and free‐radical scavenging ability (the DPPH assay) per dry matter of the green asparagus bud segment and butt segment

	Phenolic content (mg GAE/ g dry matter)	Free‐radical scavenging ability (mg TE/ g dry matter)
Bud segment	8.423 ± 1.159	12.560 ± 0.449
Butt segment	4.338 ± 0.430	6.564 ± 0.274

### 3.2 Effect of microwave power on the solution temperature

**Figure 2:**
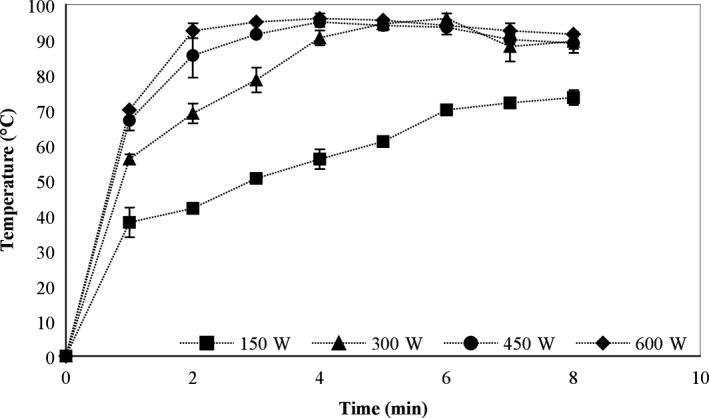
Temperature progress of the water blanching solution during microwave treatment

Figure 2 displays the temperature change of the blanching solution (water) at different microwave power. The results showed that the microwave power is proportional to the heating rate in first 4 min. After 4‐min heating by microwave, water temperatures corresponding to 150, 300, 450, and 600 W power were 56, 90.5, 95 and 96°C, respectively. At higher microwave power (300, 450, and 600 W), prolonging the exposure to microwave past 4 min causes almost no change in the temperature, and eventually, a slight temperature decrease. On the other hand, heating at 150 W showed a relatively proportional relationship of temperature to time. However, at this microwave power, maximum temperature, reached after 8 min of heating, only achieved 73°C, which is significantly lower than temperature at other power level.

In this study, the increase in heating rate was proportional to the microwave power in the first 4 min of heating. This result may be attributed to nature of microwave, which is electromagnetic waves carrying energy. To be specific, when the microwave interacts with water or polar molecules, it rotates molecules and releases heat (Rostagno & Prado, 2013), inducing greater heat conversion and in turn, increased heating rate. However, after 4 min of heating at 300, 450, and 600 W where the temperature has already approximated the boiling point (100°C at 1 atm), the absorbed energy might have been diverted into shifting the phase from liquid to vapor state, therefore maintaining the stable solution temperature (88–96°C). This is contrast with case of heating 150 W where the solution temperature was only 57°C after 4 min of heating since the absorbed energy had been mainly used to give out heat in solution.

### 3.3 Effects of microwave blanching time

#### 3.3.1 Effects of microwave blanching time on sensory

The texture and color changes in asparagus butt segment after microwave blanching at 300W for different durations were recorded and displayed in Table 2. It was found that in samples, which were treated for more than 4 min, the color and texture of the material had been significantly altered. After 8 min of blanching, the green color of sample became darker, the tissue texture was softer, and the fluid was released out from the surface of the sample. This could be explained by high temperature, resulted microwave irradiation. As shown from Figure 2, heating at 300 W for 4 min had already elevated the temperature of blanching solution to approximately 90°C, allowing possibilities of reactions including polyphenol oxidation and chlorophyll degradation. Oxidation of polyphenol produces o‐quinones that act as a browning pigment, making the sample color darker. Chlorophyll is the main colorant in fruit and vegetable with green color. Destruction of this pigment also contributes to color change of the sample during blanching. In addition, chemical changes such as solubilization and depolymerization of pectic polysaccharides could affect the compositions of cell walls, causing expansion and breakdown of plant structure and in turn changes in toughness of fruits and vegetables (Nisha, Singhal, & Pandit, 2006). However, softer materials also makes further processes such as drying, and extraction become easier as it could reduce drying time and improve yield of extraction.

**Table 2:** Changes in texture and color of asparagus butt segment by time after blanching microwave at 300 W

	Fresh	2 min	4 min	6 min	8 min
Picture	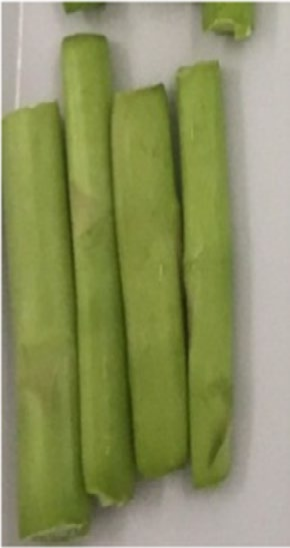	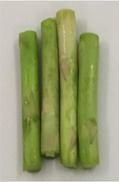	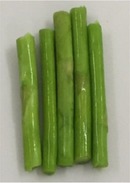	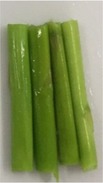	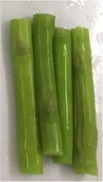
Texture	Tough, not bruised	Tough	The outer part is soft	soft	Soft, the outer skin is wrinkled
Color	green	Light green, same as fresh samples	Vivid green, shiny	Green with light yellow tint	Withering green

#### 3.3.2 Effects of microwave blanching time on the percent retention of phenolics and antioxidant capacity

Table 3 showed the percent retention of phenolics and antioxidant capacity of asparagus butt segment at different blanching times by microwave. Results indicated that the percent retention of phenolics decreased significantly from 83.34% ± 2.83 at 2‐min blanching to 53.43% ± 1.03 at 8‐min blanching (*p* < .05), which is consistent with results from a previous report (Jaiswal, Gupta, & Abu‐Ghannam, 2012). This result can be explained as after blanching at 300 W for 2 min, and the temperature of blanching solution reached 70°C (Figure 2) that broke down the phenolic compounds into simple isomers by polyphenol oxidase activity. Furthermore, losses in phenolic content could also be caused by diffusion into the blanching solution and self‐degradation.

**Table 3:** The percent retention of phenolics and antioxidant capacity of asparagus butt segment at different blanching time by microwave at 300 W

Blanching time (min)	Total phenolic retention (%)	Free‐radical scavenging ability retention (%)
2	83.34 (2.83) ^a^	69.35 (7.91) ^a^
4	72.47 (2.13) ^b^	65.52 (4.27) ^a^
6	59.88 (2.74) ^c^	56.74 (8.23) ^a^
8	53.43 (1.03) ^d^	31.20 (2.90) ^b^

Note

Data are expressed as mean (standard deviation) and values within a column with the same letter are not significantly different (*p* > .05)

For antioxidant capacity, overall, the free‐radical scavenging ability tended to decrease significantly with time. The retention percentage of DPPH power was insignificantly different between 2‐min blanching sample and 4‐min blanching sample (*p* > .05). In comparison with the fresh sample, the loss of antioxidant capacity of the blanched sample after 4 min was approximately 35 percentage points. The free‐radical scavenging ability at 6 and 8 min of blanching was respectively lower by 9.77 and 25.55 percentage points compared to that at 4 min. Thus, the rise in blanching time decreased the antioxidant capacity of samples because antioxidant compounds were subjected to the degradation over time. In this study, the retention of phenolics was proportional to the residual percentage of antioxidant capacity. Other studies also found that there is a positive correlation between phenolic content and antioxidant activity in spinach (Amin, Norazaidah, & Hainida, 2006) and broccoli (Zhang & Hamauzu, 2004).

### 3.4 Effects of microwave power

#### 3.4.1 Effects of microwave power on sensory characteristics

Texture and color of the asparagus butt segment with regards to the microwave power are described in Table 4. The results showed that after being blanched at 150 W, the hardness and the bright green color of the fresh sample remained unchanged. This is because the wavelength at this power supply provides a low value of 150 J. At microwave power of 150 W, temperature in the sample is at 56°C, which is inadequate to cause noticeable damage to the cell wall as well as the color components of the green asparagus butt segment.

**Table 4:** Changes in texture and color of asparagus butt segment after blanching at different microwave powers for 4 min

	Fresh	150W	300W	450W	600W
Picture	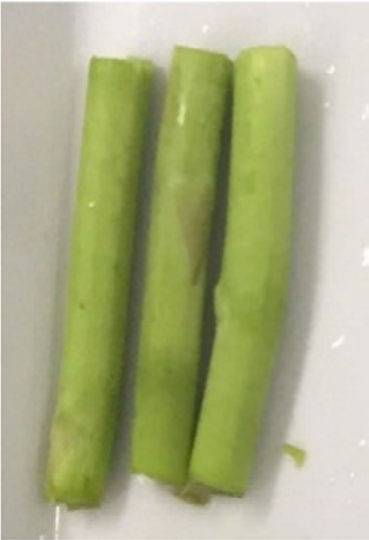	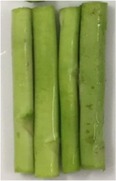	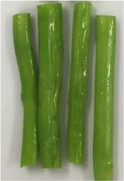	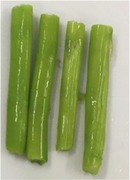	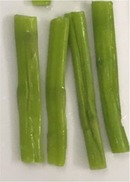
Texture	Tough, not bruised	Tough	Soft, wrinkles in the outer shell	Soft, dehydrated, wrinkle	Soft, dehydrated, wrinkle
Color	Characteristic slight green	Slight green like fresh samples	Vivid green	Green with light yellow tint	Yellow tint

As the samples were blanched at 300, 450, and 600 W, the asparagus butt segment becomes softer and the wrinkles were observed at the epidermis layer. Moreover, the initial green color of the material became darker due to the detriment of chlorophyll at high temperature. In addition, high temperatures could promote cell wall disruption, cell depletion, and the formation of the wrinkles in the epidermis layer. These results are commensurate with another kinetics study of asparagus. According to Weemaes, Ooms, Van Loey and Hendrickx (1999), green pigments (chlorophyll) decomposed when being heated at temperature of above 80°C. In addition, the pigment content suffered a loss of 3.2% with every one‐minute exposure to microwave of 650 W (Muftugil, 1986). Brewer and Begum (2003) also found that different radiation at microwave power of 385 W, 490 W, and 700 W for 4 min significantly reduced the brightness (L* value) of the sample (Brewer & Begum, 2003).

#### 3.4.2 Effect of microwave power on the percent retention of phenolics and antioxidant capacity

Table 5 shows the changes in retention of polyphenols and antioxidant capacity with respect to microwave power. For total phenolics, content of compound at 150 W was 126.33% ± 8.77 and tended to decrease when microwave power was higher than 150 W. The dramatic increase in polyphenols content at 150 W can be explained by the protein denaturation of the carotenoids‐protein complex found in chloroplasts. In some studies, the phenolics (including tannin and nontannin) content of green asparagus was found to be increasing by approximately 23% after heat treatment (Fanasca et al., 2009). This may be due to the inactivation of the polyphenol oxidase enzyme during the precipitation process leading to polyphenol degradation (Yamaguchi et al., 2003). The increase in microwave capacity elevates electrical energy absorbed into the solution and the raw material. This causes an increase in the motion of the polar molecules and in turn the temperature of the solution, thus promoting the decomposition reaction. Previous disclosures indicate that increased temperatures and blanching times may lead to the loss of phytochemicals in cabbage (Jaiswal et al., 2012), carrots, green beans, broccoli (Patras, Tiwari, & Brunton, 2011), and green asparagus (Drinkwater, Tsao, Liu, Defelice, & Wolyn, 2015; Fuentes‐Alventosa, Jaramillo‐Carmona, et al., 2009). For antioxidant capacity, the results in Table 5 showed that antioxidant activity was 76.99% ± 6.57 at 150 W and there was no striking difference in retention values at different microwave power except for at 600 W, where the retention value reached the lowest point at 58.27% ± 2.68. This suggests that as microwave power increases, the ability to capture free radicals decreases. The phenomenon can be explained by the linear correlation between polyphenol content and antioxidant capacity. As mentioned in Table 5, the retention percentage of polyphenol content was maximized at 150 W. On the other hand, the temperature of 58°C generated by this capacity is not sufficient to induce decomposition of antioxidant compounds. In addition, the carotene compounds (~ 25 μg/g dry weight) (García‐Herrera, Sánchez‐Mata, Cámara, Tardío, & Olmedilla‐Alonso, 2014) in the material under microwave treatment can be transformed into antioxidant products. At 600 W, the retention percentage decreased by 12.4% compared with that at 450 W. This can be explained by the degradation of phenolic compounds or other compounds that have antioxidant properties, caused by high temperature of higher than 90°C. Another study by Papetti, Daglia and Gazzani (2002) showed that the free‐radicals scavenging ability in vegetables decreases when exposed to various types of thermal treatment, such as blanching. These results are consistent with Wu et al. (2004), who found that the antioxidant capacity of broccoli and carrot after cooking decreased by 22% and 67%, respectively (Wu et al., 2004). The percentage of nutrient loss and antioxidant capacity depends on the type of material, the structure of the product (Ferracane et al., 2008), or the extraction solvent used (Sultana, Anwar, & Ashraf, 2009).

**Table 5:** The percent retention of phenolics and antioxidant capacity of asparagus butt segment at different blanching microwave power for 4 min

Microwave power (W)	Total phenolic retention (%)	Free‐radical scavenging ability retention (%)
150	126.33 (8.77) ^a^	76.99 (6.57) ^a^
300	73.26 (3.01) ^b^	70.67 (2.48) ^ab^
450	69.25 (0.51) ^b^	71.93 (7.42) ^ab^
600	57.15 (1.15) ^c^	58.27 (2.68) ^b^

Note

Data are expressed as mean (standard deviation) and values within a column with the same letter are not significantly different (*P* > .05)

## 4 CONCLUSIONS

This study has investigated effects of blanching time and microwave power on sensory characteristics, phenolics, and antioxidant capacity of green asparagus butt segment. Results showed that blanching time and microwave power significantly influenced sensory value, total phenolics, and antioxidant capacity of green asparagus butt segment. Longer blanching time or the higher microwave power caused darker color, softer texture, reduced total phenolics content, and antioxidant capacity. For total phenolics content and antioxidant capacity, the results showed that highest of DPPH radical scavenging ability and highest of total phenolic content were obtained in the sample blanched at 300 W in 4 min. Although the sample blanched at 150 W in 4 min retained 126.33% of total phenolic content, the temperature of blanching solution at this blanching condition only reached 57°C, which might be insufficient to inactivate enzymes in fruits and vegetables, such as polyphenol oxidase and peroxidase, causing greater quality loss during processing or storage. Therefore, appropriate parameters for microwave blanching of the green asparagus butt segment are 300 W for 4 min. For further study, degradation kinetics of total phenolics degradation, antioxidant ability, and quality properties should be investigated to allow better processing control for manufacture of food products from the green asparagus butt segment.

## CONFLICT OF INTEREST

All authors declare no conflict of interest with regard to the described research, the publication of the result, and financial issues.

## AUTHORS CONTRIBUTION

Investigation, Thi Yen Nhi Tran; Supervision, Long Giang Bach; Writing – original draft, Thi Van Linh Nguyen; Writing – review & editing, Duy Chinh Nguyen.

## ETHICAL APPROVAL

This study does not involve any human or animal testing.
